# Topological Jackiw-Rebbi states in photonic Van der Waals heterostructures

**DOI:** 10.1038/s41377-026-02392-5

**Published:** 2026-07-19

**Authors:** Sam A. Randerson, Paul Bouteyre, Xuerong Hu, Oscar J. Palma Chaundler, Alexander J. Knight, Casey K. Cheung, Roman Gorbachev, Kenji Watanabe, Takashi Taniguchi, Yue Wang, Helgi Sigurðsson, Alexander I. Tartakovskii

**Affiliations:** 1 School of Mathematical and Physical Sciences, The University of Sheffield, Sheffield, S3 7RH UK; 2 Department of Physics and Astronomy, The University of Manchester, Oxford Road, Manchester, M13 9PL UK; 3 National Graphene Institute, The University of Manchester, Oxford Road, Manchester, M13 9PL UK; 4 Research Center for Electronic and Optical Materials, National Institute for Materials Science, 1-1 Namiki, Tsukuba, 305-0044 Japan; 5 Research Center for Materials Nanoarchitectonics, National Institute for Materials Science, 1-1 Namiki, Tsukuba, 305-0044 Japan; 6 School of Physics Engineering and Technology, University of York, York, YO10 5DD UK; 7 Institute of Experimental Physics, Faculty of Physics, University of Warsaw, ul. Pasteura 5, PL-02-093, Warsaw, Poland

**Keywords:** Photonic crystals, Nanophotonics and plasmonics, Photonic devices, Imaging and sensing

## Abstract

Topological phenomena, first studied in solid state physics, have seen increased interest for applications in nanophotonics owing to highly controllable light confinement with inherent robustness to defects. Photonic crystals can be designed to host topologically protected interface states for directional light transport, localization and robust lasing via tuning of the bulk topological invariant. At the same time, van der Waals (vdW) materials, in both their monolayer and quasi-bulk forms, emerge as exciting additions to the field of nanophotonics, with a range of unique optoelectronic properties and intrinsic adherence to any type of host material, allowing fabrication of complex multi-layer structures. We present here a 1D topological photonic platform made from stacked nanostructured and planar layers of quasi-bulk WS_2_ to achieve Jackiw-Rebbi (JR) interface states between two topologically distinct gratings in the near-infrared range around 750 nm. Such states are measured in the far-field using angle-resolved reflectance contrast spectroscopy and exhibit linewidth of 10 meV and highly directional emission with an angular bandwidth of 8.0^°^. Subsequent local mapping of the structure via sub-wavelength resolution scattering-type scanning near-field optical microscopy (s-SNOM) reveals strong spatial confinement of the JR state to the interface region between the gratings. Finally, we couple in the JR state the photoluminescence of monolayer WSe_2_ incorporated in a five-layer vdW grating heterostructure, giving rise to directional enhancement of the excitonic emission of up to 22 times that of uncoupled monolayer, thus demonstrating the potential of the topological interface states for highly directional light emission in addition to light scattering.

## Abstract (web summary)

Topological van der Waals WS_2_ photonic gratings host confined Jackiw-Rebbi states, yielding directional modes and 22x enhanced WSe_2_ monolayer emission.

## Graphical abstract

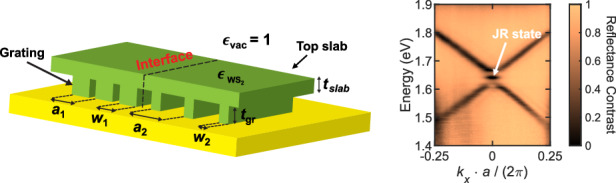

## Introduction

Topological photonics is a rapidly evolving field promising intrinsically robust control of light at the nanoscale compared to conventional nanophotonic systems, where performance varies greatly with fabrication quality. Stemming from the discovery of topological insulators in 1980 via the quantum Hall effect^1^, optical analogs have been more recently investigated in the form of topologically-protected photonic interface states, for efficient waveguiding and light manipulation with inherent immunity to back-scatter^2^. For instance, 2D spin Hall photonic lattices have been realized using silicon, yielding confined interface states with unidirectional propagation selected by the polarization handedness of the incident light^3,4^. Such topologically-protected resonances were measured in the far-field, but can also be directly probed in the near-field via scanning near-field optical microscopy (SNOM) methods^5,6^.

Of closer importance to this work, one-dimensional topological photonic crystals^7^ have been studied to realize highly localized interface states between systems of different Zak phases^8^. Such states manifest within the leaky radiation continuum as Jackiw-Rebbi (JR) interface states^9^, characterized by robust and directional emission situated at the center of the photonic band gap in energy. The history of JR states can be traced back to a solution of the 1D Dirac equation, where fermions of opposing mass form highly localized interface states regardless of small changes in the masses^9^. Via comparison to the Su-Schrieffer-Heeger (SSH) model^10^, photonic JR interface states have been identified between grating structures with opposing Zak phases in simulation^11,12^ and experiment^6,13–15^, as well as in dielectric particle arrays^16,17^. Such JR interface states boast applications such as lasing^16,18^ and beam shaping^12,15^, therefore providing additional, topologically-protected channels for advanced control of light on the nanoscale.

Layered 2D crystals, known as van der Waals (vdW) materials, have also garnered significant interest over the last two decades owing to their inherent interlayer attractive forces, and unique optoelectronic properties that undergo large changes between bulk and monolayer^19,20^. For example, monolayers of transition metal dichalcogenides (TMDs), a prominent member of the vdW family, exhibit direct band gaps with high oscillator strength excitons, yielding efficient photoluminescence (PL)^21,22^. Their bulk counterparts are emerging as capable and versatile candidates for sub-wavelength light confinement and control^23,24^, owing to high refractive indices with uniaxial anisotropy, and low losses over a large portion of the visible wavelength range^25^. TMDs and other vdW materials can be readily fabricated by hand via mechanical exfoliation, adhering easily to a wide range of substrates without the need for chemical bonding or lattice matching. This allows for incorporation of TMD monolayers onto silicon-based structures and, for example, achieving spin Hall topological interface states^26^ and optical valley Hall currents^27^ for exciton-polaritons. Furthermore, standard lithography and etching techniques can be successfully applied to vdW materials to realize high quality patterned structures in experiment^28–30^ such as 2D photonic crystals etched from bulk TMDs, observed to host unidirectional propagating interface states^31^. Van der Waals materials thus present exciting additions to nanophotonics and light-matter physics, aiding where specific optoelectronic properties are required, or advanced multi-layer heterostructures need to be built.

We note that current experimental studies on JR states in 1D gratings primarily consider low index materials such as SiN for the grating device, hence requiring large structures of several microns in thickness and near micron periods to operate within the near-IR range^15^. More compact structures fabricated from low index TiO_2_ suffer from greatly reduced contrast of the mode dispersions, and require additional ion-assisted deposition steps^14^. Here, we achieve high contrast grating modes and topological JR interface states in much more compact structures ( < 100 nm thickness), fabricated using high index WS_2_ (*n*_WS2_ = 4)^25^ via simple mechanical exfoliation and “pick-and-place” polymer stamp transfer techniques of up to five individual planar layers, to operate within the near-IR range around 750 nm.

**Fig. 1:**
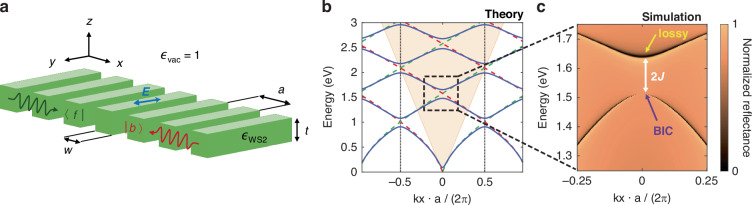
Theoretical model of coupled grating modes. **a** Schematic of suspended WS_2_ grating with parameters *a*, *t*, and *w* corresponding to the grating period, thickness, and width of each WS_2_ beam respectively. Incident plane wave is polarized parallel to the grooves (i.e. along *y*) denoted by the blue double arrow. 〈*f|* and *|b*〉 correspond to the forwards and backwards propagating grating modes respectively, traveling perpendicular to the grooves. **b** Grating mode dispersion calculated using a simplified model for coupled forward and backwards propagating guided modes (denoted by the green and red dashed lines respectively). The first Brillouin zone is denoted by the vertical black lines. Modes are band folded into the first Brillouin zone and exist within the light cone (orange shaded region). Blue curves correspond to the energies of diffractively coupled guided modes, showing gap opening at branch crossings. **c** Normalized angle-resolved reflectance simulated using RCWA for a suspended WS_2_ grating showing emergence of a lossy mode and infinitely narrow linewidth BIC on the upper and lower energy branches respectively

In this work we begin by outlining the analytical model of our system, defining the Hamiltonian for propagating modes within a 1D grating to achieve a bound state in the continuum (BIC) and lossy mode, split by a band gap at the Γ-point in the momentum space (see Fig. 1). Topological effects of the band structures are further considered via comparison to the 1D Dirac equation, where combining gratings with opposite topological phases results in the formation of a localized JR interface state. We then detail the design and rationale behind our inverted gratings, where an additional high index WS_2_ slab is simulated on top of a WS_2_ grating to promote band inversion and a topological phase transition upon tuning the filling factor (FF). Leveraging on the inherent interlayer attractive forces of TMDs, we realize such inverted gratings via polymer-stamp transfer of bulk WS_2_ flakes onto prefabricated double grating devices. Through this, we experimentally demonstrate precise tuning of the photonic band gap and band inversion via the control of the refractive index contrast in the grating akin to Refs. ^11,32^, with subsequent observation of photonic JR states between topologically distinct structures in the visible-to-near-infrared regime. The far-field responses of all resonances are fully characterized with angle-resolved reflectance contrast throughout momentum space, with the JR state exhibiting an angular bandwidth of 8.0^°^, along with a 10 meV linewidth in energy. We verify the strong spatial localization of the JR state by direct probing via scattering-type SNOM (s-SNOM). Finally, PL from an hBN-encapsulated WSe_2_ monolayer within a five-layer double grating heterostructure is coupled to the JR interface state, yielding highly directional emission up to 22 times stronger than in uncoupled monolayer.

Altogether, we realize several conceptually similar structures, which were fabricated with unavoidable experimental defects owing to the complexity of the fabrication, including exfoliation, patterning, and transfer steps of up to five 2D layers of various thicknesses. Nevertheless, we find that each of these structures exhibits a JR edge state near the expected mid-gap energy. This experimental observation is in agreement with computational tests of the topological robustness of the JR edge states in 1D lattices in Ref. ^11^ where several structures were considered in which both the filling factor and lattice period were randomly varied.

## Results

### Theoretical model for guided modes of photonic gratings

Assuming continuous translational symmetry in the lateral *y* direction, the periodic modulation of the grating acts on the guided photonic modes through a potential operator of the form *V* = *u*(*x*)*w*(*z*), where *u*(*x*) = *u*(*x* + *a*) is along the grating period and *w*(*z*) is a step function along the vertical direction as illustrated schematically in Fig. 1a. Counter-propagating guided electromagnetic modes along *x* that differ by an integer number of the primitive reciprocal lattice number *K* = 2*π/a* are diffractively coupled and exhibit Bragg reflection. Replicas of the guided mode dispersion fold across the Brillouin zone with a gap opening around their crossing point as calculated using a simplified model considering the coupling between dispersive modes in Fig. 1b. For example, second order diffractive coupling between modes *|e*^*±iqx*^〉 with the wavevectors *q* = *±K* + *k*_*x*_, where *k*_*x*_ ≪ *K*, opens a gap at the Γ-point which corresponds to a second order stop band. Here, we will focus on the transverse electric (TE) modes, whose dispersion is approximately given by *ω*_*±K*_ = *ω*_*K*_ *± vk* with the group velocity *v* around *q* = *K*. As the guided modes *|e*^*±iKx*^〉 fold into the Brillouin zone, they also couple with lossy modes residing at normal incidence *q* = 0 within the light cone (shaded orange region) through a first order diffraction process^33^. Sufficiently close to the crossing point (see boxed region of Fig. 1b) the dynamics of photons in the grating can be described by a non-Hermitian Dirac-like Hamiltonian^11,33,34^,

1 $$\widehat{H}=\left(\begin{array}{cc}v{k}_{x} & J{e}^{i\phi}\\ J{e}^{-i\phi} & -v{k}_{x}\end{array}\right)-i\gamma \left(\begin{array}{cc}1 & 1\\ 1 & 1\end{array}\right)$$

Here, we set *ω*_*K*_ = 0 without loss of generality. The first term contains the Hermitian diffractive coupling parameter *Je*^*iϕ*^ between counter-propagating modes resulting in a gap of size 2 *J*, as in Fig. 1c. The second anti-Hermitian term describes coupling *γ* of the modes to the lossy radiative continuum through a Friedrich-Wintgen type process^35^ (see Supplementary Note 1 for more details). Thus, in Fig. 1c the linewidths of the modes at k = 0 are 2*γ* and 0 for the lossy and BIC modes, respectively.

The mirror symmetry of the grating, taken to be at *x* = 0 so that *u*(*x*) = *u*(*-x*), implies that *ϕ* ∈ {0*, π*}, and guarantees the presence of a symmetry protected photonic BIC at the Γ-point in the antisymmetric energy branch^36^. It is worth noting that the BIC is also topologically tied to a polarization vortex in the far-field^37^. The phase *ϕ* of the diffractive coupling mechanism can be flipped from 0 → *π* by adjusting the pitch and filling factor of the grating^11,33,34^. Physically, this phase flip corresponds to inverting the energy hierarchy between the symmetric and antisymmetric standing wave Bloch states.

The eigenvalues of Eq. (1) describe the dispersion of the guided photons at low momenta, corresponding to the angle-resolved reflectance simulation (see “Methods”) shown in Fig. 1c using the Rigorous Coupled-Wave Analysis (RCWA) technique,

2 $${\omega }_{\pm }\left({k}_{x}\right)=-i\gamma \pm \sqrt{{\left(v{k}_{x}\right)}^{2}+{J}^{2}-{\gamma }^{2}-2{iJ}\gamma \cos (\phi )}$$

The eigenvectors are derived in Supplementary Note 1. Here, *ω*_*±*_ refer to the upper (+) and lower (*-*) energy branches (bands) of the grating standing waves. The diffractive coupling *J* ≫ *γ* is the main parameter responsible for the bandgap opening at *k*_*x*_ = 0. When *ϕ* = 0, a BIC mode of an infinite lifetime appears in the center of the lower *ω*_*-*_ antisymmetric branch while a lossy 2*γ* mode appears in the upper symmetric *ω*_+_ branch (see Fig. 1c). If *ϕ* = *π*, equivalent to *J* → *-J*, band inversion takes place and the antisymmetric state containing the BIC is now higher in energy while the symmetric state is lower. The presence of the BIC at *k*_*x*_ = 0 can be appreciated from,

3 $${\rm{Im}}\left[{\omega }_{\pm }(0)\right]\approx -\gamma [1\pm \cos (\phi )]$$

### Theoretical model for photonic Jackiw-Rebbi interface state

Our photonic guided mode Hamiltonian Eq. (1) can be transformed into a spinless one-dimensional non-Hermitian Dirac equation with a mass term *m* through the unitary transformation $\widehat{U}=\left({\widehat{\sigma }}_{x}+{\widehat{\sigma }}_{z}\right)/\sqrt{2}$,

4 $${\widehat{U}}^{\dagger }\widehat{H}\widehat{U}={\widehat{H}}_{{\rm{Dir}}}=c{\widehat{\sigma }}_{x}{\widehat{p}}_{x}-{\widehat{\sigma }}_{z}m{c}^{2}-i\gamma$$

Here, *p*_*x*_ = ℏ*k*_*x*_, *c* = *v* and *m* = ℏ(*J* cos(*ϕ*) *- iγ*)*/v*^2^. It has been known for some time in relativistic quantum field theory that the 1D Dirac equation hosts a topologically protected localized state with fractional particle numbers known as a Jackiw-Rebbi mid-gap state^9,38^. Formally, the JR state appears spatially at the interface of two Dirac systems with opposite mass signs *m*(*x*) = *m*_0_(2*H*(*x*)*-*1) where *H*(*x*) is the Heaviside function, and the state wavefunction is written

5 $$\left|{\psi }_{{\rm{JR}}}\right\rangle =\sqrt{\frac{c{m}_{0}}{2{\rm{\hbar }}}}{e}^{-{c|m}(x)\cdot {x|}/\hbar }\left({1}\atop{i}\right)$$

The JR state is energetically positioned exactly in the center of the gap of the Dirac Hamiltonian. Its topological origin can be appreciated from the fact that no energy mismatch is imposed on the structure. That is, flipping the sign of *m* does not alter the eigenvalues in Eq. (4), precluding the presence of trivial bound states. A photonic JR state can be constructed if two gratings that differ by *π* in their diffractive coupling phase *ϕ* are put together^11^. The interface between the two gratings corresponds then to a jump *J* → *-J* which can be expressed in terms of the complex mass,

6 $$m(x)=\frac{\hbar }{{v}^{2}}[J(2H(x)-1)-i\gamma ]$$

The topological origin of the JR state can be traced back to its condensed matter analog in the electron bands of 1D chains of conjugated polymers, the SSH model^10,38^. Therein, the topological invariant is known as the quantized Zak phase ${\mathcal{Z}}=i{\int }_{\mathrm{BZ}}\,\left\langle {\psi }_{\pm }\right|{\partial }_{k}\left|{\psi }_{\pm }\right\rangle {dk}\in \{0,\pi \}$ ^8^ where |*ψ*_*±*_〉 are the symmetric and antisymmetric Bloch modes. The integral is over the Brillouin zone and 〈*ψ*_*±*_*|∂*_*k*_ | *ψ*_*±*_〉 is integrated over the real space unit cell. In the photonic grating, *ϕ* = 0 *→ π* corresponds to ${\mathcal{Z}}$ = 0 *→ π* implying that the band inversion represents a topological phase transition which invokes the bulk-boundary correspondence with consequent presence of a midgap JR state. The existence of the JR state and the associated Zak phase mismatch can also be linked to the surface impedance condition *Z*_*L*_ + *Z*_*R*_ = 0, where *Z*_*L,R*_ are the surface impedances of the left and right grating at the interface^39^. Moreover, the band inversion can also be linked to a different type of a topological invariant in the far-field, given by the half-integer charge of an optical Skyrmion number^40^.

### Design and fabrication of inverted double-grating structure

**Fig. 2:**
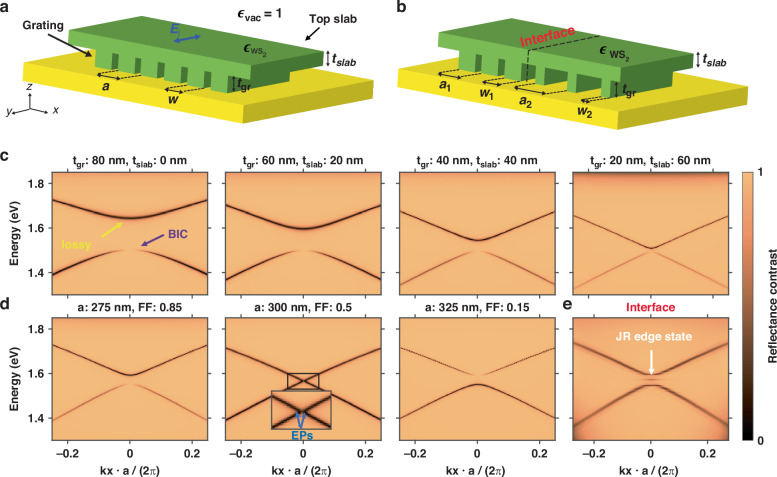
Simulated angle-resolved reflectance contrast of WS_2_ single and double inverted grating structures on gold. **a** Schematic of the single inverted grating structure. *a*, *w*, *t*_gr_, and *t*_slab_ correspond to the grating period, width of WS_2_ beams, and thicknesses of the grating and slab respectively. *ϵ*_WS2_ and *ϵ*_vac_ correspond to the WS_2_ and vacuum permittivities respectively. Blue double arrow denotes incident polarization direction ***E***_i_ parallel to the grooves of the grating, corresponding to TE excitation. **b** Schematic of the double inverted grating structure with respective periods and widths *a*_1,2_ and *w*_1,2_. Interface between the two gratings denoted by the black dashed line. **c** Simulated reflectance contrast of single WS_2_ grating on gold with increasing thicknesses of *t*_slab_ from left to right leading to reduction of the band gap. Total thickness of the structure is kept constant for each. **d** Simulated tuning of the grating filling factor to achieve photonic band inversion. The period is also slightly adjusted to shift each mid-gap position to the same energy. The three panels show reflectance contrast for single inverted gratings. Left panel shows BIC on the lower energy branch for a high FF. Center panel exhibits two exceptional points (EPs)^41^ where the modes cross for FF = 0.5. Right panel shows BIC flipped to the upper energy branch for low FF. **e** Simulated reflectance contrast of a double inverted grating structure combining the high and low FF gratings as in (**b**). A topologically protected JR interface state is observed at the mid-gap energy as a result

Here we explain the rationale behind the unique design of our grating structures and detail the subsequent fabrication process. As per our analytical model presented above, to obtain photonic band inversion and thus a Jackiw-Rebbi interface state, one needs to tune the grating filling factor to close the photonic gap and reopen it. However, the high refractive index of a WS_2_ grating leads to a band gap so large, that no amount of tuning of the filling factor can close the gap (see Supplementary Note 2). For this reason, we employ an additional WS_2_ flake on top of our gratings to controllably reduce the effective refractive index contrast between the etched and unetched sections of the gratings, thus reducing the photonic band gap. This design also leverages on the intrinsic vdW adhesive forces of such materials, allowing simple transfer of the top bulk WS_2_ flake (i.e. slab) onto any prefabricated nanostructures and devices as illustrated schematically in Fig. 2a. Here, a gold substrate was chosen owing to its strong reflectivity, thus yielding high contrast with the optical grating modes in reflectance measurements. In addition, gold further illustrates the range of substrates compatible with vdW materials, whilst also acting as a natural etch stop to produce as-designed structures. We note that plasmonic effects are not expected owing to the s-polarized incident light used to excite only TE grating modes.

The effects of adding a top WS_2_ slab to the gratings were simulated using the RCWA technique (see “Methods”) as shown in Fig. 2c. The electric field component of the incident light was fixed along the direction parallel to the grooves (i.e. along *y* in Fig. 2a), corresponding to TE polarization. Here, the angle-resolved reflectance contrast with respect to the wavevector component *k*_*x*_ from the inverted grating structure is plotted while changing top slab thickness *t*_slab_ and keeping the total structure thickness constant. A clear reduction of the band gap is observed with increasing *t*_slab_, corresponding to a decrease in the effective refractive index contrast between the repeating high and low index sections of the structure.

To initiate photonic band inversion, we then simulated a grating with a top slab with parameters *t*_gr_ = 35 nm and *t*_slab_ = 45 and varied the FF as in Fig. 2d. The FFs of the gratings were chosen to obtain two different topological regimes through a band inversion mechanism. The left panel shows a grating structure with a BIC on the lower energy branch. By continuously decreasing the filling factor, the gap is reduced until it closes and forms two exceptional points (EPs)^41^, non-Hermitian equivalent of a Dirac point, as seen in the middle panel. In the context of our 2 *×* 2 model given in Eq. (1), these points appear when *J* = 0. By further decreasing the filling factor, the gap reopens, as seen in the right panel with the BIC on the upper energy branch, leading to the change of the topological nature of the structure. Here, the grating period was also tuned to compensate for the redshift induced by the change of the filling factor, thus ensuring that the mid-gap energy was the same for each structure. Using this inverted grating design, we show that photonic band inversion via closing and re-opening of the band gap is possible. To obtain the JR interface state, we then placed the high and low filling factor gratings from the left and right panels of Fig. 2d adjacent to one another, as illustrated by the schematic in Fig. 2b. Via further reflectance contrast simulations, this time using a Finite-Difference Time-Domain (FDTD) solver (see “Methods”), a clear state within the photonic band gap was realized as in Fig. 2e, which we attribute to a topologically protected JR interface state.

**Fig. 3:**
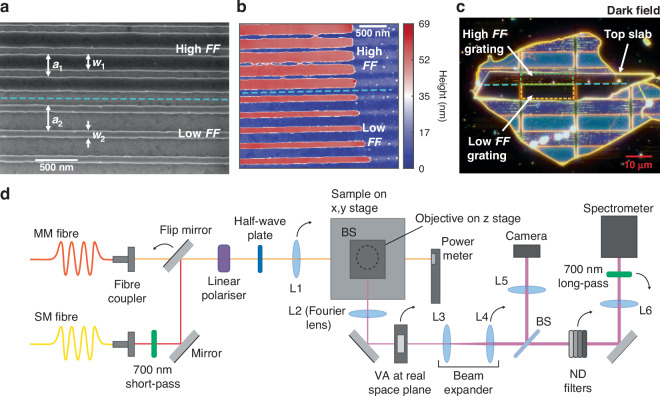
WS_2_ double grating structure fabrication and experimental Fourier setup. **a** SEM image of example double grating interface before top slab transfer. Dashed light blue line corresponds to the interface between high and low FF gratings at the top and bottom of the image respectively. **b** AFM image of double grating interface before top slab transfer. Measured grating thickness 47 nm. **c** Dark field microscope image of WS_2_ inverted double grating structure with parameters *t*_gr_ = 47 nm, *t*_slab_ = 41 nm, *a*_1,2_ = 279, 319 nm, *F*_1,2_ = 0.81, 0.41. High and low filling factor inverted gratings denoted by green and orange dotted boxes respectively. **d** Schematic of Fourier spectroscopy setup with angle-resolved reflectance and PL capability. All optics on flip mounts denoted by curved arrows. Sample sits on the *x, y* stage beneath the beam splitter (BS) and objective (denoted by black dashed circle) with numerical aperture (NA) 0.7. For reflectance measurements the multi-mode (MM) fiber is coupled to a white light source with long-pass filter removed. Lens L1 ensures uniform Köhler illumination of the sample, whilst L2 images the back focal plane (i.e. Fourier plane) of the objective. Variable aperture (VA) allows selection of signal region from the real-space plane. L3 and L4 act as a beam expander for the Fourier spot. L5 and L6 focus the Fourier plane onto the sample camera and spectrometer slit respectively. For PL the single-mode (SM) fiber is coupled to a 637 nm laser with both short- and long-pass filters in the optical path and L1 removed

To realize such inverted double-grating structures in experiment, we began by mechanically exfoliating WS_2_ flakes of a range of thickness (*∼*10–500 nm) onto electron-beam evaporated gold on silicon wafers (see “Methods”). Subsequent electron-beam lithography of the substrates coated with a positive resist produced a pattern of alternating high and low filling factor grating pairs. The grating period and electron beam dosage were varied across multiple pairs to account for different flake thicknesses and etching rates. Reactive ion etching using a partially chemical (SF_6_) and physical (CHF_3_) etch recipe produced grating structures etched to the gold with parallel beams of WS_2_. The interface between high and low filling factor gratings were imaged via scanning electron microscopy (SEM) and atomic force microscopy (AFM) as in Fig. 3a, b, respectively, for characterization of the fabricated period, filling factor, and thickness.

We note that with such thin layers of material used ( < 50 nm), partial etching down to nanometer resolution required to achieve band inversion at the same mid-gap energy would be challenging to realize experimentally. A way around this issue is through our inverted grating design, with both the grating and unetched top flake thicknesses chosen with nanometer precision, thus allowing reliable tuning of the photonic modes.

The transfer step involved first a calibration via simulating the reflectance contrast spectra of the gratings with the measured parameters upon varying top slab thickness. This process enabled accurate determination of the slab thickness required to achieve two topologically distinct adjacent gratings, and thus a JR interface state. WS_2_ was then exfoliated onto PMMA spin-coated on silicon covered with a PVA sacrificial layer. A flake of the required thickness was selected with the help of AFM and transferred onto the gratings, covering the interface (see details in “Methods”). The resulting inverted double-grating structures on gold were imaged via dark field microscopy as shown in Fig. 3c. Here, the top slab was of thickness *t*_slab_ = 41 nm and the grating had *t*_gr_ = 47 nm. The darker blue rectangles of the etched bottom flake correspond to the high filling factor gratings with a period *a*_1_ = 279 nm and a filling factor *F*_1_ = 0.81, and the lighter blue rectangles correspond to the lower filling factors gratings with *a*_2_ = 319 nm and *F*_2_ = 0.41. The top WS_2_ slab was positioned to cover at least one of each type of grating, as well as the interface between them for measurement of all three topological regimes. The covered high and low filling factor grating regions (i.e. inverted gratings) of interest are denoted by the green and orange dotted boxes in Fig. 3c, respectively.

### Far-field measurements of Jackiw-Rebbi interface state

**Fig. 4:**
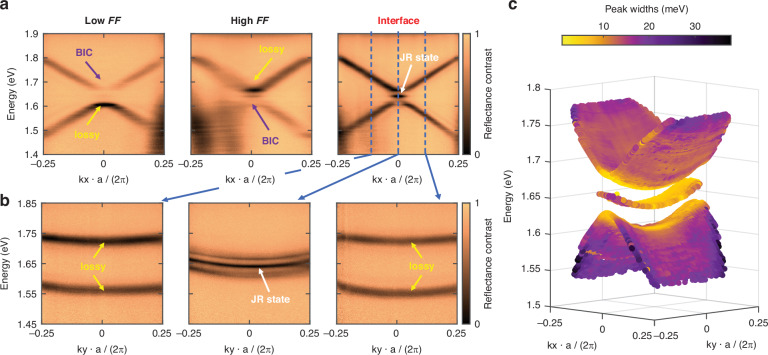
Experimental reflectance contrast characterization of the JR interface state. **a** Angle-resolved reflectance contrast measurements of the low FF grating (*a*_2_ = 330 nm, FF_2_ = 0.3), high FF grating (*a*_1_ = 261 nm, FF_1_ = 0.7), and whole structure including the interface respectively, in the *k*_*x*_ direction at *k*_*y*_ = 0 µm^*–*1^. Wavevector normalized to the respective grating periods *a*, where the average between the high and low FF gratings is taken for the interface region (right panel). **b** Angle-resolved reflectance contrast of the grating interface region with respect to the *k*_*y*_ direction for three different values of *k*_*x*_, as denoted by the blue dashed lines in the right panel of (**a**). **c** Three-dimensional tomographic reconstruction of the grating and interface state modes in momentum space

Angle-resolved reflectivity contrast measurements of the structure were obtained using a Fourier spectroscopy setup as depicted schematically in Fig. 3d. A white light illumination source was used with TE configuration to compare to the simulated spectra (more details in “Methods”). The reflectivity contrast maps in the *k*_*x*_ direction (for *k*_*y*_ = 0) of the low FF grating region, interface, and high FF grating region are presented in Fig. 4a. In agreement with our previous simulations from Fig. 2d, one can observe a BIC on the high energy branch of the spectra from the left panel of Fig. 4a corresponding to the low FF grating, and a (quasi-) BIC flipped to the low energy branch for the high FF grating in the central panel of Fig. 4a. Here we observe a quasi-BIC at *k*_*x*_ = 0 as the top WS_2_ slab did not fully cover the entire high FF grating (green dotted box in Fig. 3c), thus leading to a finite linewidth and obscured reflectance at negative *k*_*x*_. Additional experimental data from a separate structure are presented in Supplementary Note 3 exhibiting true BICs for both the high and low FF gratings, whilst also highlighting the repeatability of our transfer fabrication method, and tunability of the bands in energy. By measuring at the interface region shown in the right panel of Fig. 4a, there is a clear feature at the mid-gap energy, not present in the two gratings alone, corresponding to a JR state. These experimental results therefore not only confirm the successful photonic band inversion via tuning of the filling factor, but also the different topological phases of such inverted band structures, resulting in the formation of an interface-localized Jackiw-Rebbi state within the band gap.

We further considered the reflectance contrast along the *k*_*y*_ direction by rotating the sample whilst keeping the TE excitation configuration as in Fig. 4b. Three measurements were taken at the interface region for three different values of *k*_*x*_ as denoted by the dashed blue lines in the right panel of Fig. 4a. For all three sets of reflectance contrast spectra, we observe that the dispersion of the two lossy grating modes is parabolic with positive curvature. As *k*_*x*_ is reduced to zero, the JR state emerges and follows the parabolic dispersions of the two lossy modes, remaining at the mid-gap energy with positive curvature in the *k*_*y*_ direction. Via fitting to a Lorentzian function, we extract a linewidth of 10 meV, and further calculate that the JR state yields an angular emission bandwidth in the direction perpendicular to the grooves of just ∆*θ*_*x*_ = 8^°^, yet can be in- and out-coupled throughout the full measurement region in the direction parallel to the grooves with ∆*θ*_*y*_ = 88^°^ (±44°, the maximum angle range for an 0.7NA objective). These results therefore highlight the highly confined nature of the JR interface state in TMD-based inverted double-gratings, with strong localization in both energy and momentum space.

We subsequently plot the full 3D reconstruction of the experimental grating mode structure in *k*_*x,y*_ in Fig. 4c. One can observe that the lower energy mode has a saddle shape, with negative curvature in *k*_*x*_ but positive curvature in *k*_*y*_. In contrast, the higher energy mode displays a 3D parabolic shape with positive curvature in both directions, but greater curvature in *k*_*x*_. In addition, the JR state is a parabolic stripe along *k*_*y*_, with a width of 1.1 µm^*–*1^ in *k*_*x*_. As well as being able to visualize the full dispersion of all modes in momentum space, this three-dimensional reconstruction also highlights the relative linewidths of each mode in energy. We expect the BICs at the band edges and JR state to be most strongly confined to the structure for *k*_*x*_ = 0, owing to the symmetry conditions required to host such states^42,43^. This can be observed experimentally via the narrower linewidths of the modes along the line *k*_*x*_ = 0, but also shows that even at large angles in the direction parallel to the grooves up to *±*44^°^ from the normal, the linewidths of the resulting modes change negligibly. Such 1D double grating structures therefore possess highly selective directivity profiles depending on the plane of incidence.

### Near-field measurements of Jackiw-Rebbi interface state

From our RCWA simulations and experimental angle-resolved reflectivity measurements, the existence of a topological photonic JR interface state in WS_2_ double inverted grating structures is clearly demonstrated in the far-field. We now investigate the near-field localization of this state through both simulation, and experimental probing of the local electric field distribution via s-SNOM.

**Fig. 5:**
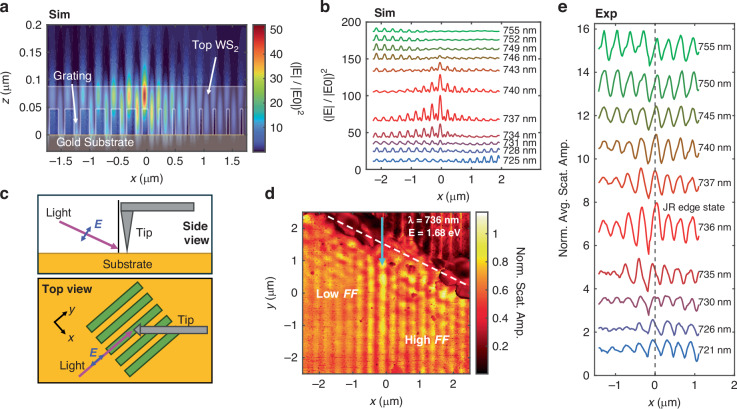
Near-field study of the JR interface state. **a** FDTD simulation of the near-field distribution of the JR state for 60^°^ incident illumination angle. **b** Profiles of the simulated electric field strength along the top surface of the structure for changing incident wavelength. **c** Schematic of the s-SNOM setup from side (top panel) and top-down (bottom panel) views. Incident light depicted by the pink arrow propagating at 60^°^ to the tip axis. Blue double arrow denotes polarization direction (p-polarized) with component along the tip axis. The grating is oriented parallel to the propagation direction of the incident light allowing to excite the edge mode along the *k*_*y*_ direction. **d** Experimental normalized near-field scattering amplitude image at 736 nm excitation of the WS_2_ double grating structure. Light blue arrow highlights interface between low and high FF gratings where near-field enhancement is observed. Dashed white light corresponds to edge of the top WS_2_ slab. **e** Averaged near-field scattering amplitude profiles over multiple scanning paths displaced in the y direction for varying incident wavelength. Grating interface centered at *x* = 0 showing enhancement only around 736 nm illumination

Figure 5a depicts the simulated electric field confinement in a cross-sectional slice through the middle of the double gratings as calculated via FDTD. To emulate an s-SNOM measurement, the incident excitation was a p-polarized plane wave traveling at 60^°^ to the substrate normal, in the direction parallel to the grooves (see “Methods”). It is clear that the JR state is strongly localized at the interface between the two topologically distinct gratings, with an up to 50 times enhancement of the incident wave intensity. In addition, we observe that confinement is highest within the top WS_2_ slab rather than the grating itself, which we attribute to the higher overall effective refractive index of the slab portion. The field also protrudes significantly out of the top of the structure, enabling direct probing via a nanoscale tip.

We then simulated a range of illumination wavelengths, and plot profiles of the electric field strength along the top surface of the structure, i.e. where we are able to probe with an s-SNOM tip, as in Fig. 5b. These simulations show that the JR state peaks in electric field confinement at around 737 nm (1.68 eV). We also note that at lower wavelengths, the overall field strength is higher to the right of the grating interface (*x* = 0) which corresponds to the lossy mode of the high FF grating, whereas the fields are weaker to the left of the interface corresponding to the BIC. This behavior flips as we tune through the JR state to higher wavelengths owing to the band inversion between the high and low filling factor gratings.

To successfully probe the JR interface state via experimental s-SNOM measurements, the sample had to be precisely orientated owing to its strong directivity. With the high incident illumination angle to the substrate normal (see s-SNOM “Methods”), excitation of the JR state was only possible via light propagating in the plane of incidence parallel to the grooves of the grating, as depicted by the pink arrow in Fig. 5c. This illumination angle corresponds to zero wavevector in the *k*_*x*_ direction, with mode dispersion along *k*_*y*_ given by the central panel of Fig. 4b. We further extrapolated the JR state dispersion in *k*_*y*_ up to the incident angle of 60^°^ (see Supplementary Note 4), yielding an estimated excitation energy of 1.68 eV (735–738 nm), which agrees perfectly with our simulations from Fig. 5b. Importantly, this excitation regime means a component of the incident electric field lies parallel to the grooves as shown by the blue double arrow in Fig. 5c, which is required to excite the TE-polarized grating modes and thus the JR interface state.

Following careful sample alignment, we performed s-SNOM measurement at 736 nm excitation as in Fig. 5d and observed enhanced scattering from the interface between the two gratings as marked by the light blue arrow. The data shown have been median line levelled separately either side of the grating interface to minimize the intrinsic material response, which differs depending on the effective refractive index experienced by the tip. We thus focus on scattering arising mostly from probing the localized electric fields associated with the grating modes, as described in more detail in Supplementary Note 5.

We then repeated the scan for a range of wavelengths and plotted line profiles of the scattering intensity averaged over multiple rows in the *y* direction as in Fig. 5e, where the grating interface and thus JR state is centered at *x* = 0. We observe that the peak in scattering at 736 nm along the dashed line quickly decays when changing the incident wavelength, which correlates very well with our electric field profile simulations. The localization of the enhanced scattering exactly where we expect it spatially, also at the correct energy we predicted from our far-field measurements for light incident at 60^°^, strongly suggests that we have successfully probed the near-field response of this topological photonic JR interface state, confirming its highly localized nature in real space, *k*-space, and energy.

### Photoluminescence enhancement at the Jackiw-Rebbi state

**Fig. 6:**
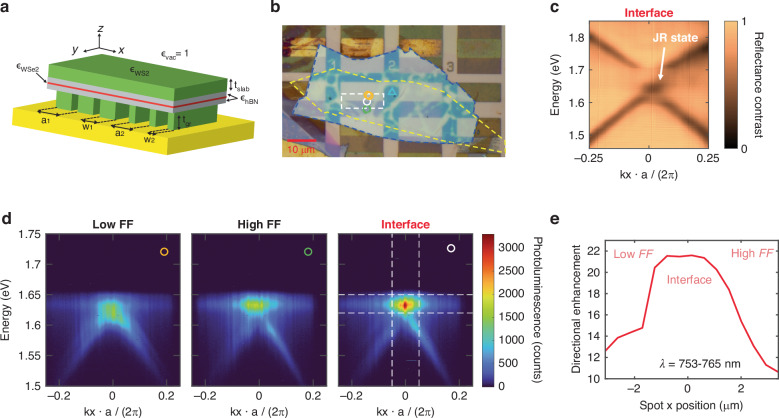
Photoluminescence enhancement via active grating heterostructure. **a** Schematic of the active double grating heterostructure composed of hBN-encapsulated WSe_2_ between a WS_2_ double grating and top slab on a gold substrate. **b** Optical microscope image of the fabricated heterostructure. Top WS_2_ slab and monolayer WSe_2_ outlined by dashed blue and yellow lines respectively. White dashed box corresponds to Fourier RC measurement region. Colored circles correspond to the Fourier PL excitation spots. Light blue triangle denotes the Fourier PL reference measurement location on encapsulated monolayer WSe_2_ away from the gratings. **c** Angle-resolved RC taken from the white dashed region in (**b**) around the double grating interface showing JR state within the photonic band gap. **d** Angle-resolved photoluminescence measurement of the low FF grating, high FF grating and the interface between the two corresponding to the orange, green, and white circles from (**b**) respectively. **e** Photoluminescence directional enhancement factor along the direction perpendicular to the grooves compared to uncoupled monolayer. Signal integrated over *k*_*x*_ and energy as shown by the dashed white lines in (**d**)

After demonstrating the near-field localization of the JR interface state in both simulation and experiment, we now study the PL enhancement and directivity of an emitter coupled to such a state. To do so, we consider a similar double grating structure shown schematically in Fig. 6a, in which an hBN-encapsulated WSe_2_ monolayer is embedded between the grating layer and the top WS_2_ slab. The thin hBN layers prevent charge transfer between the WSe_2_ monolayer and bulk WS_2_ with negligible effect on the grating mode structure, thus enabling bright PL emission. A combination of PDMS and PMMA-based transfer techniques was used to build this structure as explained in more detail in “Methods”. Figure 6b presents an optical microscopy image of the fabricated double-grating heterostructure, in which the top WS_2_ flake and encapsulated WSe_2_ monolayer are indicated by the blue and yellow dashed lines respectively. Here we used a top flake of thickness *t*_slab_ = 32 nm with a grating *t*_gr_ = 69 nm. The top and bottom hBN layers were of 3 and 6 nm thicknesses, respectively. To confirm the presence of a JR interface state in this more complex heterostructure, we performed angle-resolved reflectance contrast measurements as with the previous sample. Figure 6c shows results from the grating interface, taken from the white dashed rectangular region of Fig. 6b using the variable aperture as detailed in the “Methods” section. A clear JR state is once again visible within the photonic band gap, shifted slightly to lower energy likely owing to coupling with the WSe_2_ exciton at 1.65 eV.

PL measurements were performed at room temperature using the Fourier setup from Fig. 3d, with the introduction of a 637 nm laser for excitation. The measurements were taken from three different locations indicated by the circles in Fig. 6b, corresponding to the two separate low and high filling factor gratings, and the grating interface. Clear coupling of the WSe_2_ exciton PL to the lossy grating mode can be seen in the left panel of Fig. 6d for the low FF grating, where enhanced signal follows the dispersion of the modes as measured via angle-resolved reflectance contrast in Fig. 6c. In the center panel, a dip in PL can be observed around *k*_*x*_ = 0 at *∼*1.6 eV, corresponding to emission unable to outcouple to the far-field from the BIC within the high FF grating. Some enhancement is also observed around 1.64 eV, however this corresponds to leaked signal from the JR state owing to the close spatial proximity of the measurement regions, and the finite extent of the JR state electric field profile. Finally, the right panel of Fig. 6d shows the strongest PL enhancement from the grating interface region at the JR state energy. The PL is enhanced over the same range of *k*_*x*_ as the JR state in reflectance contrast, therefore indicating coupling of the WSe_2_ excitonic emission to the state.

To quantify the directional PL enhancement from monolayer WSe_2_ coupled to the JR state, the PL intensity was integrated over the region depicted by the dashed white lines in the right panel of Fig. 6d. The size of this integration region is equal to the fitted linewidth of the JR state-coupled PL peak in both *k*_*x*_ and energy. This integration was repeated for PL dispersions measured at multiple *x* positions (vertical direction in Fig. 6b) and then divided by the integrated counts from a PL measurement taken at a reference position as marked by the light blue triangle in Fig. 6b. In this region, the bottom WS_2_ remains unetched, resulting in an hBN-encapsulated monolayer WSe_2_ embedded between two WS_2_ slabs (as detailed further in Supplementary Note 6), which are transparent at the WSe_2_ emission energy of 1.65 eV. The reference PL emission was therefore not coupled to any of the grating or interface modes, with no dispersion in *k*-space (see Supplementary Note 6). As such, the directional enhancement factor corresponds to the ratio of PL from the grating heterostructure between 753*-*765 nm wavelength emitted into a wavevector range of *±*1.1 µm^*-*1^ (angular bandwidth of *±*7.5^°^), to that of the PL from the reference region over the same wavelength and wavevector range. Note that the wider angular bandwidth for the active grating heterostructure compared to the passive device presented in Fig. 4a is likely owing to losses induced by the monolayer WSe_2_, and the presence of hBN which reduces the overall effective refractive index and thus broadens the modes. The directional enhancement results are plotted in Fig. 6e and give an indication of how much additional emission is directed upwards of the sample plane via JR state coupling compared to the omnidirectional emission from uncoupled monolayer WSe_2_. We calculate a directional enhancement factor of between 11 and 13 for the monolayer coupled to the separate grating modes, and up to 22 for the JR state-coupled emission at the grating interface.

## Discussion

Here we discuss some of the figures of merit of optical cavities and compare them with the characteristics of the studied JR edge states. We summarize this discussion in Table 1 in Supplementary Note 7.

We note that the primary advantage of the JR state lies in its topological origin rather than pure modal confinement. Furthermore, unlike traditional Fabry-Perot (FP) and photonic crystal (PhC) cavities where the free spectral range (FSR) is constrained by the cavity size *L* (FSR*∝* 1*/L*), the JR state is a mid-gap state whose spectral isolation is determined by the bulk photonic bandgap. This decoupling allows for single-mode operation over larger spatial areas, facilitating better overlap with exfoliated 2D materials, particularly compared with PhC cavities, where further constraints arise from limitations of fabrication as most PhC cavities are realized in suspended membranes^44^.

In the approach for realization of the JR states reported in our work (a grating covered with an unetched slab), the 2D material monolayer is integrated within the structure (“monolithic” integration), as opposed to the surface deposition and interaction with the PhC cavity mode through the evanescent field. This presents another advantage of our devices over PhCs.

From the calculations of the JR wave-function we estimate a lateral confinement length of the JR state as *L* ≈ 600 nm, comparable with the lateral confinement in L3 PhC cavities^44^. The vertical confinement for both the JR state and the PhC cavity mode is defined by the thickness of the photonic structure, roughly of the order of 100 nm. FP cavities embedded between high reflectivity distributed Bragg reflectors (DBRs) are usually at least *λ/*2*n* in size (*λ* is the cavity mode wavelength and *n* is the refractive index of the cavity material) with the mode extending in the DBRs leading to the reduced vertical mode confinement of a few 100 s nm and the lateral mode confinement of a few microns in micropillars^44^.

The *Q*-factor of the studied JR edge state of *≈*150 is lower than in typical PhC defect cavities. For example, *Q* ≈ 2000 was achieved in suspended hBN membranes^45^, *Q* of up to a few 10^4^ can be achieved experimentally in III-V semiconductor PhCs containing quantum dots^44^, while in silicon photonic nano-structures, optimized for telecommunications wavelengths where the absorption is negligible, it reaches a few 10^6^^44^.

We note that the quality of the WS_2_ crystal may also influence the results, mostly due to the absorption below the bandgap. However, many recent ellipsometry measurements show negligible absorption in WS_2_ for wavelength >700 nm in commercially sourced material. See for example ellipsometry results for WS_2_ in refs. ^23,25,46^.

The 1D photonic lattices studied in this work provide a very rich band-structure with high-order BIC and bright states that can be tuned independently by varying the slab thickness and the parameters of the lattice. The symmetry of the lattice can be broken or new symmetries introduced in technically feasible ways, thus introducing additional control of the modes and their coupling to light^47^, similar to much more complex 2D metasurfaces^48^.

While 1D photonic lattices provide access to localized edge states, the exploration of the physics of both guided modes and edge states in 1D lattices is still far from exhausted with many possibilities for the novel photonic design approaches and potential applications. Recent reports include hybridization of two JR edge states^14^, strong coupling of JR states with perovskite excitons providing foundation for JR-based polariton lasing^13^. High directionality of emission from a JR edge state can be of interest for privacy screen applications requiring photonic modes highly directional in one dimension only^12^.

In 2D topological structures, conceptually similar approach is used for realization of the 1D edge states (in contrast to the localized JR states in 1D lattices such as in this work), see e.g. our recent work on realization of *Z*_2_ topological photonic insulators made from WS_2_ in ref. ^31^ and references to a broader field therein. In comparison with 1D lattices, 2D structures exploiting 1D edge states require much more complex nanofabrication and generally use smaller features that must be precisely controlled in two dimensions and reproduced on the scale of at least several tens of microns. Propagation of 1D edge modes is further compromised by the material losses that are difficult or impossible to control via nanofabrication.

Further to 1D edge states, localized topological mid-gap states can be created in 2D structures^49^. The single mid-gap mode in the Dirac-vortex topological cavity is the photonic realization of the Jackiw-Rossi zero mode^50^. The Dirac-vortex topological cavities are a new type of optical micro-resonators that utilize a 2D honeycomb photonic crystal with a generalized Kekulé modulation to create a vortex bandgap^49^. One of the advantages of this system is an unprecedentedly large FSR leading to robust single-mode operation, overcoming the traditional trade-off between resonator size and mode spacing^49^.

In conclusion, we realized here precise tuning and band inversion of 1D photonic grating modes in stacked van der Waals devices, yielding topologically-protected Jackiw-Rebbi interface states in both simulation and experiment with strong theoretical agreement. Via far-field angle-resolved reflectance spectroscopy, we fully characterized the grating mode dispersions throughout *k*_*x,y*_, revealing strongly directional emission of the JR state along the direction perpendicular to the grooves, with an angular bandwidth of 8^°^ and linewidth 10 meV. Such JR states also exhibit clear spatial confinement around the boundary between topologically distinct gratings as detected using scattering-type scanning near-field optical microscopy. We further fabricated an active five-layer double grating heterostructure incorporating hBN-encapsulated monolayer WSe_2_, demonstrating directional enhancement of the exciton PL via coupling to the JR state. Our results thus highlight the utility of vdW materials for easily integrable and adaptable multi-layer on-chip devices, able to enhance and shape the radiation patterns of coupled emitters, with in-built topological protection.

## Materials and methods

### Simulations

Angle-resolved reflectance simulations of single gratings were performed using an open source Python implementation of the Rigorous Coupled-Wave Analysis (RCWA) technique named *S*^4^ ^51^. A unit cell was defined with periodicity of the grating, composed of stacked layers for the substrate, grating, top slab, and superstrate. Experimentally measured WS_2_ anisotropic refractive index data was used from ref. ^25^. The experimentally measured refractive index data for gold was taken from ref. ^52^. The grating layer was imprinted with a pattern according to the desired filling factor to achieve a periodically modulated structure. TE polarization was considered for the excitation source, with incident angle varied in the direction perpendicular to the grooves (i.e. *x*). Reflected light was subsequently normalized to a reference simulation of just the substrate to achieve reflectance contrast.

Double gratings were simulated using the Finite-Difference Time-Domain (FDTD) method through the commercial software package Lumerical. 20 periods of each adjacent grating were simulated to ensure accuracy, with the simulation region encompassing a 2D slice through the cross-section of the structure. Plane wave source with TE polarization was swept over a range of incident angles and reflected light intensity measured, as with the RCWA simulations. Perfectly matched layer (PML) boundaries were used along all directions to reduce any reflections at the simulation edges. Electric field profiles taken using frequency-domain field and power monitors.

### Fabrication

WS_2_ flakes from HQ graphene were mechanically exfoliated onto electron-beam evaporated 150 nm gold + 10 nm Ti on Si substrates. 105 ^°^C temperature used to promote adhesion, before cooling and then exfoliating. A positive resist (ARP-9 AllResist GmbH) was then spun onto the substrates at 3500 rpm for 60 s, before heating for 2 minutes at 180 ^°^C. Electron beam lithography was then performed using a Raith GmbH Voyager system at 50 kV accelerating voltage and 560 pA beam current, producing patterns of alternating high and low filling factor grating pairs. Multiple pairs with varying electron beam dosage and period were patterned onto each flake to account for different thicknesses and variations in etch rates. Reactive ion etching for 40 s with 0.14 mbar pressure and a DC bias of 135 V was used with a partially chemical and physical etch recipe of both SF_6_ and CHF_3_ gases. This produced parallel beams of WS_2_ without a preferential zigzag etch, as with purely chemical etching. Subsequent layers of multi-layer hBN (NIMS) and WS_2_ for transfer were exfoliated onto PMMA-coated Si substrates with a PVA sacrificial layer between. Flakes of ideal thickness were identified via atomic force microscopy, before scribing of a circle *∼*3 mm diameter around the desired flake. The PVA was then dissolved with deionized water, and the floating membrane ‘fished’ with a metal ringed cantilever. Inside a nitrogen-environment glovebox, the membrane was then brought into contact with the desired grating via a 3D stage with vacuum arm and heated to 180^°^C to melt the PMMA. Acetone/IPA washing followed by a brief O_2_-plasma cleaning removed the remaining membrane, leaving flakes adhered to the gratings. Monolayer WSe_2_ (HQ Graphene) was exfoliated onto PDMS stamps and transferred using the same glovebox transfer setup. 65^°^C temperature was used on place-down of the stamp to promote adhesion. Reduction of the heat upon lift-off the PDMS caused detachment of the WSe_2_ from the stamp, and adherence to the target grating heterostructure sample.

### Angle-resolved reflectance

A home-built Fourier spectroscopy setup was used for experimental angle-resolved measurements of the grating structures as depicted in Fig. 3d. White light illumination was used with polarization always along the grooves of each grating (TE) dictated by a linear polarizer and half-wave plate combination. A 50:50 beam splitter subsequently redirected light to an objective lens with numerical aperture 0.7. A lens placed at its focal length from the back focal plane of the objective was used to image the Fourier plane. A variable rectangular aperture was placed at the real-space plane after the first lens to selectively collect signal from specific spatial regions of each sample. A further two-lens telescope was used to enlarge the Fourier image, before focusing onto the spectrometer slit by a final lens. Closing of the spectrometer slit to 50 µm allowed projection of a single slice in Fourier space onto the charge-coupled device (CCD), at a single *k*_*y*_ for a range of *k*_*x*_, or vice versa by rotating the sample 90^°^. As with the simulations, each grating reflectance measurement was normalized to the signal from gold substrate alone to yield reflectance contrast.

### Angle-resolved photoluminescence

The same Fourier setup was used for PL measurements by changing the excitation source to a 637 nm Vortran Stradus diode laser. Additional short- and long-pass filters were flipped into the beam path before and after the sample respectively, so that only the angle-resolved PL signal was collected by the spectrometer.

### Near-field imaging

s-SNOM data were collected under ambient conditions utilizing a neaSCOPE microscope from Attocube Systems AG/Neaspec. The neaSCOPE system was coupled with a Chameleon Compact OPO-Vis system from Coherent, consisting of a Ti:Sapphire laser and optical parametric oscillator (OPO) with a tuneable, single wavelength output selectable anywhere between 360 and 1600 nm. Light from the laser was sent into a Michelson interferometer housed within the neaSCOPE system, with an AFM in one arm of the interferometer operating under tapping mode and a clean reference mirror housed in the other. The laser light was focused onto the tip of a metal-coated AFM cantilever (ARROW-EFM from Apex Probes, PtIr coating, tapping frequency 82-85 kHz, tapping amplitude 34–37 nm, tip radius of curvature *∼*25 nm) at an angle of 60^°^ to the tip axis via a parabolic mirror. The incident polarization was aligned along the tip axis, i.e. p-polarized, thus exciting surface plasmon-polaritons at the metal/air boundary which were then used to probe the sample as it was raster scanned beneath. Further laser light scattered off the tip-sample interaction region was collected back into the interferometer before interfering with the light reflected off the reference mirror at the detector. The tapping motion of the AFM cantilever allowed the optical signal from the tip-sample interaction region to be demodulated at harmonics of the tapping frequency (order 3), thereby reducing the influence of background scattering. Compete background removal was obtained by oscillating the reference mirror at a fixed frequency, resulting in frequency mixing and the formation of side bands in the optical power spectrum that were then tracked.

## Supplementary information

Supplementary Information for Topological Jackiw-Rebbi States in Photonic Van der Waals Heterostructures

## Data Availability

The data that support the findings of this study are available from the corresponding author upon reasonable request.
