# Supplementary material for: Topological Jackiw-Rebbi states in photonic Van der Waals heterostructures
Source: Light Sci Appl. 2026 Jul 19;15:323. doi: 10.1038/s41377-026-02392-5 (PMC13380625; doi:10.1038/s41377-026-02392-5)
Supplement: Supplementary file 1 — Supplementary Information for Topological Jackiw-Rebbi States in Photonic Van der Waals Heterostructures [file 41377_2026_2392_MOESM1_ESM.pdf]

Supplementary Information for Topological Jackiw-Rebbi States in Photonic Van der Waals Heterostructures

Sam A. Randerson,[1,*] Paul Bouteyre,[1,†] Xuerong Hu,[1] Oscar J. Palma Chaundler,[1] Alexander J. Knight,[1] Casey K. Cheung,[2,3] Roman Gorbachev,[2,3] Kenji Watanabe,[4] Takashi Taniguchi,[5] Yue Wang,[6] Helgi Sigurðsson,[7] and Alexander I. Tartakovskii[1,‡]

[1] *School of Mathematical and Physical Sciences, The University of Sheffield, Sheffield S3 7RH, U.K.*
[2] *Department of Physics and Astronomy, The University of Manchester, Oxford Road, Manchester, M13 9PL, U.K.*
[3] *National Graphene Institute, The University of Manchester, Oxford Road, Manchester, M13 9PL, U.K.*
[4] *Research Center for Electronic and Optical Materials, National Institute for Materials Science, 1-1 Namiki, Tsukuba, 305-0044 Japan*
[5] *Research Center for Materials Nanoarchitectonics, National Institute for Materials Science, 1-1 Namiki, Tsukuba, 305-0044 Japan*
[6] *School of Physics Engineering and Technology, University of York, York YO10 5DD, U.K.*
[7] *Institute of Experimental Physics, Faculty of Physics, University of Warsaw, ul. Pasteura 5, PL-02-093 Warsaw, Poland*

(Dated: May 25, 2026)

* s.a.randerson@sheffield.ac.uk
† S.A. Randerson and P. Bouteyre contributed equally to this work as first authors.
‡ a.tartakovskii@sheffield.ac.uk

I. SUPPLEMENTARY NOTE 1

A more general form of the photonic Dirac Hamiltonian given in Eq. (1) in the main text reads [1],

$$\hat{H} = \begin{pmatrix} vk_x & Je^{i\phi_2} \\ Je^{-i\phi_2} & -vk_x \end{pmatrix} - i\gamma \begin{pmatrix} 1 & e^{i2\phi_1} \\ e^{-i2\phi_1} & 1 \end{pmatrix} \tag{1}$$

Here, we have explicitly written out the phases $\phi_{1,2}$ belonging to first- and second-order diffractive coupling channels which are given by their corresponding Fourier coefficients over the periodic potential $Je^{i\phi_2} = U_2 = \langle e^{i(k_x+K)x}|u(x)|e^{-i(k_x-K)x}\rangle$ and $\gamma \propto |U_1|^2$ and $\phi_1 = \arg(U_1)$ where $U_1 = \langle e^{ik_x x}|u(x)|e^{-i(k_x-K)x}\rangle$. Physically, the phases represent interference between counterpropagating modes (ϕ_2) and propagating modes with the lossy Fabry-Perot modes around normal incidence (ϕ_1). The second anti-Hermitian term in $\hat{H}$ is derived by adiabatically eliminating the dynamics of the lossy Fabry-Perot mode by assuming it decays much faster decay compared to the guided modes $|e^{-iqx}\rangle$ which are protected by total internal reflection [1]. The effective loss rate of the folded guided modes across the Γ-point is written $\gamma = |c_0|^2|U_1|^2/\gamma_0$ where $c_0 = \langle\chi^{(0)}(z)|\chi_K(z)\rangle$ describes the vertical overlap (confinement integrals) between the lossy mode $|\chi^{(0)}(z)\rangle$ and the guided mode $|\chi_K(z)\rangle$.

The mirror symmetry of the grating $u(x) = u(-x)$ implies that the Fourier coefficients are real-valued. This means that $\phi_{1,2} \in \{0, \pi\}$ and therefore $e^{2i\phi_1} = 1$ and $e^{i\phi_2} = \pm 1$. This can be understood from the Fourier expansion of the periodic potential

$$u(x) = \sum_n U_n e^{iK_n x}, \qquad K_n = \frac{2\pi n}{a}. \tag{2}$$

where the coefficients U_n belong to the Fourier transform,.

$$U_n = \frac{1}{a}\int_{-a/2}^{a/2} u(x) e^{iK_n x}\, dx. \tag{3}$$

Because $u(x) = u(-x)$ is an even function, only the cosine from the $e^{iK_n x}$ contributes which means $U_n \in \mathbb{R}$. The sign of the Fourier coefficients U_n depends on the period of the potential $u(x)$ which means that the phases $\phi_{1,2} \in \{0, \pi\}$ of the diffractive and lossy channel coupling can be flipped from $0 \to \pi$ by adjusting the pitch and filling factor of the grating [1–3]. Importantly, we always have that $e^{2i\phi_1} = 1$ which means that the presence of symmetry protected photonic bound state in the continuum (BIC) is guaranteed at the Γ-point in the antisymmetric energy branch [4] topologically tied to a polarization vortex in the far field [5].

The eigenvectors of $\hat{H}$ for $\phi = \phi_2$ and $e^{2i\phi_1} = 1$ (coinciding with our equation in the the main text) are written,

$$\boldsymbol{v}_\pm(k_x) = \frac{\pm 1}{A_\pm}\begin{pmatrix} h_\pm \\ J - i\gamma \end{pmatrix} \tag{4}$$

where $h_\pm = vk_x \pm \sqrt{(vk_x)^2 + J^2 - \gamma^2 - 2iJ\gamma\cos(\phi)}$ and $A_\pm > 0$ are normalization constants. Specifically, for $\phi = 0$ and $k_x = 0$ we have,

$$\boldsymbol{v}_\pm(0) = \frac{J - i\gamma}{\sqrt{2(J^2+\gamma^2)}}\begin{pmatrix} \pm 1 \\ 1 \end{pmatrix} \tag{5}$$

The ± here refers to a symmetric and antisymmetric standing-wave of the forward $|e^{iKx}\rangle = (1,0)^{\mathrm{T}}$ and backward $|e^{-iKx}\rangle = (0,1)^{\mathrm{T}}$ counter-propagating photons.

II. SUPPLEMENTARY NOTE 2

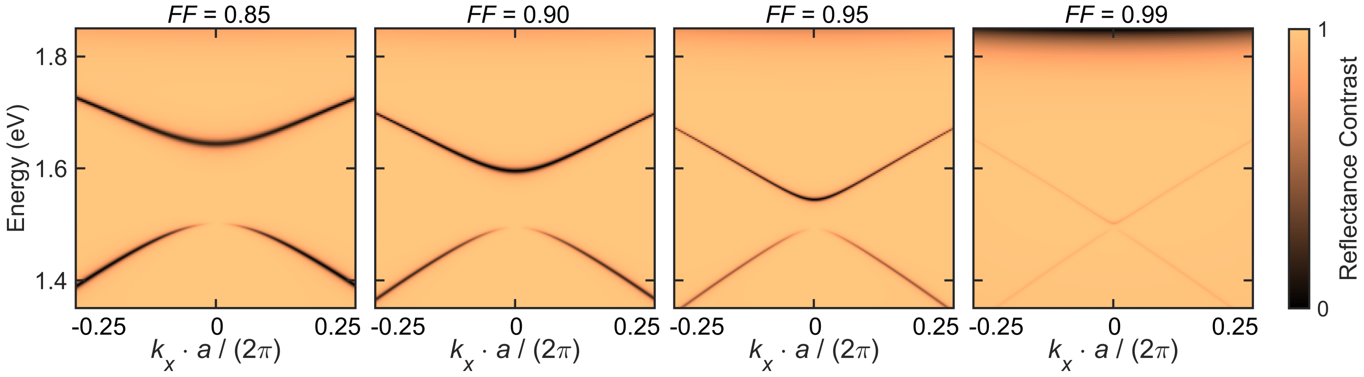

FIG. S1. **Simulated reflectance contrast of WS_2 grating on gold with changing filling factor.** From left to right: $FF = 0.85, 0.90, 0.95, 0.99$. Grating period and thickness constant for each panel with $a = 300$ nm and $t_{gr} = 80$ nm respectively. Band gap closes as FF is increased but can never be closed.

We demonstrate here the inability to fully close the associated photonic band gap using high index WS_2 gratings on gold. Figure S1 presents Rigorous Coupled-Wave Analysis (RCWA) simulations of the angle-resolved reflectance contrast from grating structures with increasing filling factor (FF). Since the refractive index of WS_2 is large (~ 4.15 [6]), the index contrast between the high and low index sections of the grating is significant, leading to a wide band gap. Increasing the filling factor in simulation tunes the coupling between the upper and lower photonic modes, thus reducing the band gap. However, as seen from Figure S1, the band gap for such structures can never fully close, no matter how high the filling factor is tuned. Besides, such large values of FF would not be experimentally feasible to realise with current nanofabrication techniques for such periods, which exhibit reduced repeatability of reactive ion etching for features < 50 nm.

To realise a photonic Jackiw-Rebbi (JR) edge state, the band gap must be fully closed and re-opened to change the topological phase [3]. Such WS_2 gratings alone are therefore not suitable for this task, which lead to the design and fabrication of the inverted gratings as detailed in the main text.

III. SUPPLEMENTARY NOTE 3

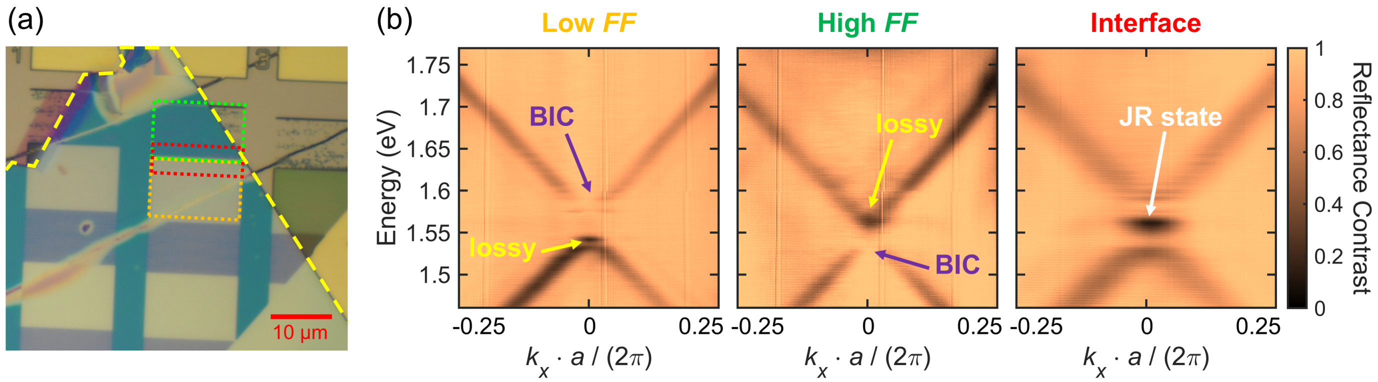

FIG. S2. **Experimental angle-resolved reflectance contrast measurements of an additional WS_2 inverted double grating sample on gold.** **(a)** Optical microscope image of the structure with transferred top WS_2 slab of thickness $t_{\text{slab}} = 44$ nm outlined by the yellow dashed line. Grating layer beneath is of thickness $t_{\text{gr}} = 52$ nm. Low FF grating denoted by the orange dotted box with period and filling factor $a_2 = 272$ nm, $F_2 = 0.84$ respectively. High FF grating denoted by the green dotted box with period and filling factor $a_1 = 356$ nm, $F_1 = 0.24$ respectively. Red dotted box corresponds to the grating interface region. **(b)** From left to right: angle-resolved reflectance contrast taken from the low FF grating, high FF grating, and interface region respectively. Clear band inversion is observed with an interface-localised JR state at the centre of the band gap.

We present here another inverted grating sample, separate to those of the main text, to prove the repeatability of obtaining JR interface states via our transfer fabrication method. An optical microscope image of the sample is presented in Figure S2 (a), with the top WS_2 slab outlined in yellow. The respective thicknesses of the grating and bulk layers are $t_{\text{gr}} = 52$ nm and $t_{\text{slab}} = 44$ nm. The low FF grating is outlined by the orange dotted box in Figure S2 (a), corresponding to the region of collected signal using the variable aperture in our Fourier setup (Figure 3 (d) of the main text). The same size collection region is used for the low and high FF gratings (green dotted box) to collect as much signal as possible. The gratings have period and filling factors $a_2 = 272$ nm, $F_2 = 0.84$ for the low FF grating, and $a_1 = 356$ nm, $F_1 = 0.24$ for the high FF grating respectively.

We subsequently measure the angle-resolved reflectance contrast for the three different regions as in the main text, and plot the results in Figure S2 (b). The left panel corresponds to signal from the low FF grating with a BIC on the upper energy branch and lossy mode on the lower branch. By tuning to high filling factor as in the central panel, there is clear inversion of the bands with the BIC on the lower energy branch. In this structure, the low and high FF grating band gaps are positioned at lower energies than the structure measured in Figure 4 of the main text, at around 1.55 eV. By measuring at the interface region we observe a clear state in the centre of the band gap, which we attribute to a topologically-protected JR interface state. Here the variable aperture used was smaller than for the separate gratings (red dotted box in Figure S2 (a)) to focus on signal from the JR state at the interface. This real-space confinement results in a slight broadening of the modes in reciprocal space and energy owing to the Heisenberg uncertainty principle.

This additional structure shows the ability to precisely and repeatably fabricate inverted double grating structures from van der Waals materials to achieve JR interface states at different energies depending on the grating parameters and flake thicknesses chosen.

IV. SUPPLEMENTARY NOTE 4

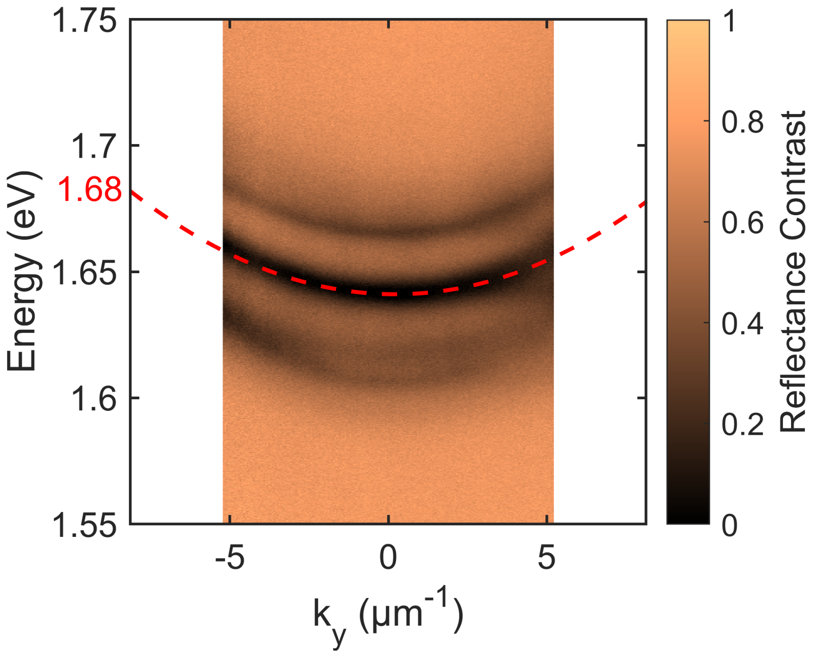

FIG. S3. **Extrapolated experimental angle-resolved reflectance contrast of interface region between WS_2 double inverted gratings on gold along k_y direction.** Upper and lower parabolic modes correspond to the grating lossy modes. Edge state dispersion fitted with a second order polynomial as shown by the red dashed line. Data extrapolated up to a wave vector corresponding to 60° incident angle.

In order to accurately determine the correct excitation energy for the Jackiw-Rebbi edge state in WS_2 double inverted gratings on gold with our scattering-type scanning near-field optical microscopy (s-SNOM) system, we extrapolated the measured dispersions from the middle panel of Figure 4 (b) of the main text. This data corresponds to angle-resolved reflectance contrast from the interface region between the high and low filling factor gratings, up to an angle of ±44° owing to our 0.7 numerical aperture objective. For the s-SNOM measurements, the incident angle was fixed at 60° to the sample normal (i.e. tip axis), and so beyond our measured range with the Fourier setup. We therefore fitted the edge state dispersion to a second order polynomial as shown by the red dashed line in Figure S3, and extrapolated up to higher wave vectors in k_y (i.e. parallel to the grating axis) corresponding to an incident excitation angle of 60°. The predicted JR state energy at this angle was 1.68 eV, which agrees perfectly with our simulations from Figure 5 (b) of the main text. In addition, performing s-SNOM scans at this energy with light propagating along the grating axis showed field localisation at the grating interface, exactly where we expect the JR state to exist as in Figures 5 (d) and (e) of the main text.

V. SUPPLEMENTARY NOTE 5

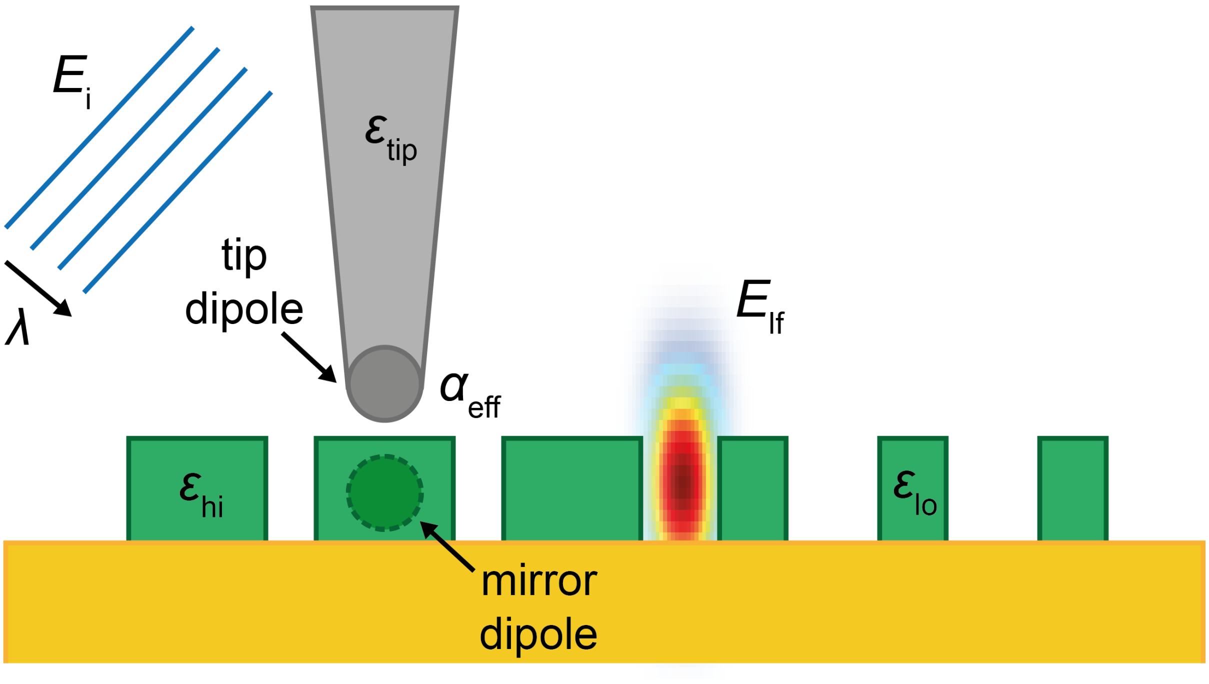

FIG. S4. **Schematic of s-SNOM probing regimes over different grating structures.** Tip dipole excited under illumination with incident field E_i, leading to corresponding mirror dipole induced within the sample. Metallic tip has permittivity ϵ_tip, and the high and low filling factor gratings having effective permittivities $\epsilon_\mathrm{hi,lo}$ respectively. α_eff represents the effective polarisability of the whole tip-sample interaction region. E_lf corresponds to the strength of local electric fields.

Since s-SNOM works via measuring the scattering intensity from the tip-sample interaction region, the measured signal is dependent upon the refractive index of the material beneath the tip at any one time. The well-known point-dipole model [7] considers a dipole excited at the apex of a metallic tip under incident illumination E_i, as depicted schematically in Figure S4. The tip dipole subsequently induces a mirror dipole within the sample beneath, with properties that depend on the sample permittivity. An overall effective polarisability of the tip-sample interaction region can thus be defined α_eff, which depends on both the permittivity of the tip and sample. The resulting scattering from this region can be expressed as $E_\mathrm{s} \propto \alpha_\mathrm{eff} E_\mathrm{i}$ [7], where $E_\mathrm{s}, E_\mathrm{i}$ are the electric fields of the scattered signal and incident light respectively. As a result, the high and low filling factor gratings will have different baseline scattering intensities owing to their different effective permittivities, as illustrated in Figure S4. Therefore, the total measured signal will be a combination of the intrinsic material properties of the sample, and the effect of the tip's interaction with locally confined electric fields E_lf, e.g. from grating and topological resonances present. To minimise the material response, we normalise the scattered signal from each different grating separately, as done in Figures 5 (d) and (e) of the main text, enabling clearer comparison of the local electric fields intensities.

There are also other processes involved, such as scattered photons from the edge of the top WS_2 slab. This region is highlighted by the white dashed line in Figure 5 (d) of the main text, where we observe straight wavefronts propagating perpendicular to the diagonal edge of the slab. We attribute this to interference between the tip-sample-scattered photons, and photons scattered from the edge of the top slab directly to the detector without interacting with the tip. The result is a pattern of dark and bright fringes that decay in intensity as the distance from the edge increases. This effect does not interact with the JR edge state directly, and simply forms either constructive or destructive interference depending on the phase mismatch with the edge-scattered photons.

VI. SUPPLEMENTARY NOTE 6

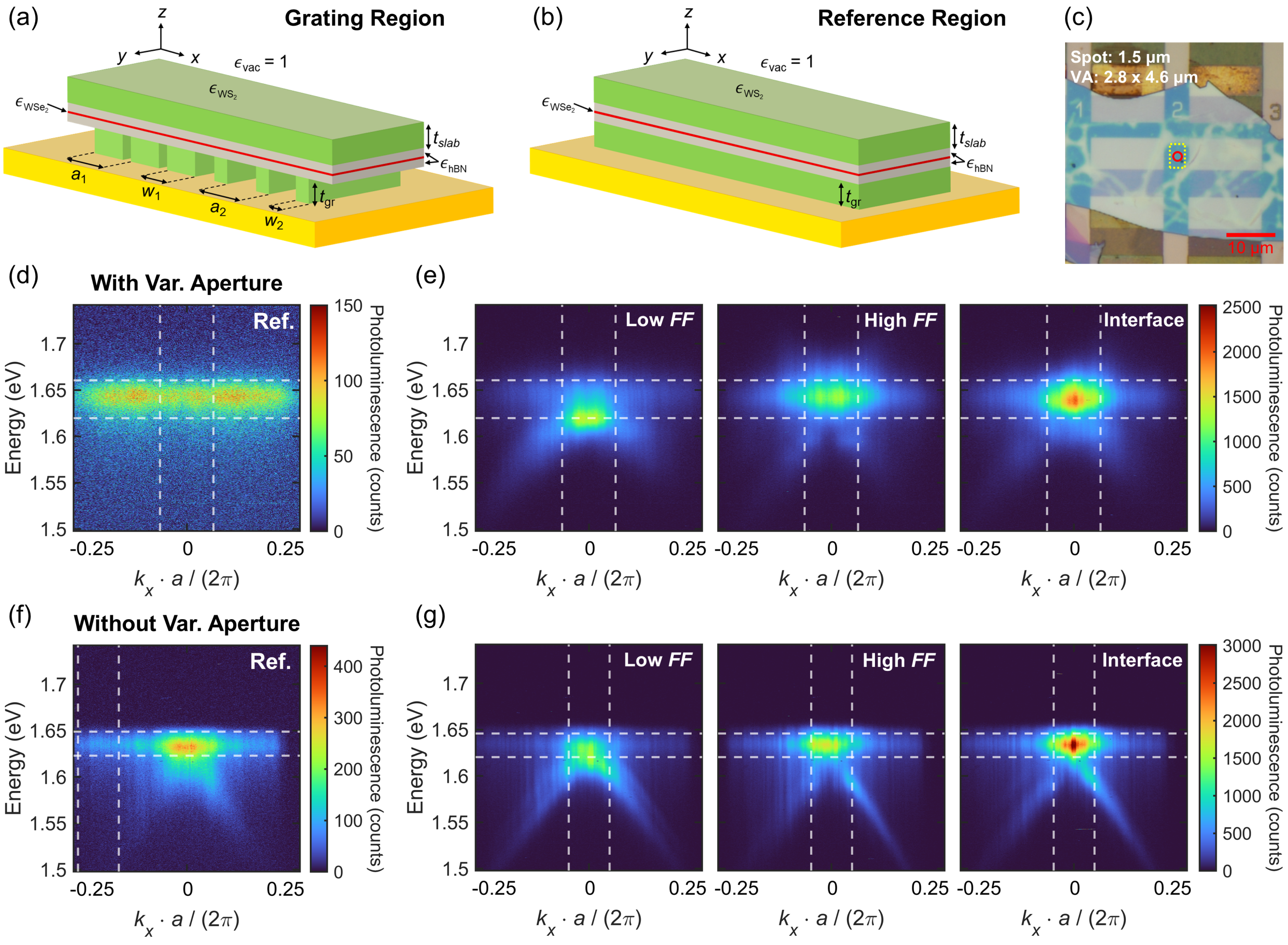

FIG. S5. **Angle-resolved photoluminescence measurements with and without variable aperture.** **(a)** Schematic of the grating heterostructure with patterned bottom WS_2 creating a double grating interface. **(b)** Schematic illustrating the structure measured at the reference position consisting of an hBN-encapsulated monolayer WSe_2 sandwiched between two unpatterned bulk WS_2 slabs on a gold substrate. **(c)** Optical microscope image of grating heterostructure where the variable aperture and laser spot correspond to the yellow dotted box and red circle respectively. Relative sizes given at the top left in white. Laser spot centred over the reference region between gratings. **(d)** Angle-resolved photoluminescence (PL) taken from the reference region depicted in **(b)** with the variable aperture illustrated by the dotted yellow box in **(c)**. No dispersion is observed across k_x with uniform PL signal around 1.64 eV corresponding to the WSe_2 excitonic emission. Dashed white lines highlight the integration region used to calculate PL directional enhancement from coupling to grating modes. **(e)** PL taken from the same positions as Figure 6 (d) of the main text with the variable aperture. Confinement in real space leads to the observed broadening of the peaks in reciprocal space and energy. **(f)** Angle-resolved PL taken from the reference region without the variable aperture. Signal is taken from the full imaged region in **(c)** leading to capturing emission from PL coupled to nearby gratings. Integration region is thus shifted left to avoid such interference. **(g)** PL taken from the same positions as Figure 6 (d) of the main text without the variable aperture. Larger collection region leads to narrower mode linewidths.

Here we detail the method used to calculate the photoluminescence directional enhancement of monolayer WSe_2 coupled to the JR interface state hosted within the grating heterostructure presented in Figure 6 of the main text. The basic principle is to first excite the structure with a 637 nm laser with spot diameter 1.5 μm, and collect the resulting PL emission over a range of wave vectors k_x using the Fourier spectroscopy setup (Figure 3 (d) of main text). A schematic of the grating heterostructure is shown in Figure S5 (a), where the excitation spot is moved perpendicular to the grooves (i.e. along x) with measurements taken at each point. We therefore collect PL coupled to both the low and high FF grating modes separately, and PL coupled to the JR state at the interface region depending on where the excitation/collection region is positioned. The PL at each x position is then integrated over k_x and energy,

and divided by a constant reference measurement to give a directional enhancement factor of the PL. The reference region is depicted schematically in Figure S5 (b), and corresponds to a region with unpatterned bottom WS_2 flake. We expect there to be no grating modes in this region and hence no coupling or modification of the PL intensity or dispersion. An optical microscope image of the measured grating heterostructure is shown in Figure S5 (c), where the red circle corresponds to the laser excitation spot positioned over the reference region between gratings. The dotted yellow box corresponds to the signal collection region taken using a rectangular variable aperture placed in the real-space plane as shown in Figure 3 (d) of the main text.

Use of the variable aperture ensured that signal only from the reference region was collected, and not leaked emission from the nearby gratings which were in close spatial proximity. The resulting angle-resolved reference PL is plotted in figure S5 (d), showing a uniform and flat dispersion at ~ 1.64 eV as expected from monolayer WSe_2. Here there are no grating modes for the excitonic emission to couple to, and thus the PL is uniform in all directions. The three panels in Figure S5 (e) from left to right correspond to angle-resolved PL measurements taken via excitation and collection from the low FF grating, high FF grating, and interface region respectively. These positions are the same as for the spectra shown in Figure 6 (d) of the main text, however here we add the variable aperture in the collection path like with the reference measurement, ensuring signal only from each of the three regions was collected. We observe similar results to Figure 6 (d) of the main text, with coupling to the lossy mode of the low FF grating (left panel), a dip in PL intensity corresponding to the BIC of the high FF grating (centre panel), and enhanced directional emission via coupling to the JR state at the grating interface (right panel). Owing to the restricted collection region of $\sim 2.8 \times 4.6$ µm from the variable aperture, the resulting peaks in the PL spectra are noticeably broader in both wave vector and energy compared to without using the aperture. This is a result of the Heisenberg uncertainty relation between real space and momentum space. For Figure 6 (d) in the main text, we therefore opted to remove the variable aperture and collect from a much larger region (whole image area shown in Figure S5 (c)) to improve mode linewidths. We note that the excitation spot still remained small at 1.5 µm diameter to excite only local modes and not the whole sample.

The dashed white lines shown in all PL spectra of Figure S5 depict the integration regions used to calculate the PL directional enhancement factors. The aim was to quantify how well the JR interface state can enhance and direct coupled PL emission normal to the sample plane compared to PL from uncoupled monolayer. We thus chose to centre the integration region around the JR state as in the right panel of Figure S5 (e), with the size of the region corresponding to the respective linewidths in both k_x and energy fitted via a Lorentzian curve. The PL directional enhancement was then simply the integrated PL over this region for each x position measured over the gratings, divided by the same integrated region for the reference region PL.

The reference PL measurement taken without the variable aperture is plotted in Figure S5 (f), and was used for the PL directional enhancement factor calculations in Figure 6 (e) of the main text. Likely owing to light propagation through the crystal to nearby gratings, we capture some coupling of the WSe_2 emission to the grating modes resulting in a weak parabolic mode dispersion. We therefore shift the integration region in k_x to a position far from any dispersions, thus avoiding enhanced signal from grating mode coupling at the reference point. We note that this method is valid since the size of the integration region remains the same, and the true dispersion of the reference region was previously shown to be uniform with respect to k_x in Figure S5 (d) using the variable aperture. Figure S5 (g) is the same as Figure 6 (d) of the main text but with the integration region highlighted for each panel for reference. There is clear enhancement of the directivity of monolayer WSe_2 PL emission when coupled to the JR state at the grating heterostructure interface region, quantified as 22 times stronger than PL from the same monolayer WSe_2 with the same pump power (216 µm) at the reference region, where the emission is uncoupled from any grating modes.

VII. SUPPLEMENTARY NOTE 7

TABLE I. Comparison of the JR edge modes with photonic modes in photonic crystal cavities and and DBR cavities

**Feature**	**Conventional PhC cavity**	**Microcavity (FP/DBR)**	**JR Edge State (this work)**
**Confinement type and mode dimensions**	Localised "defect" (geometrical), waveguide confinement 100 nm vertical, few 100 nm lateral	Reflection (vertical), few 100 nm vertical, few to few 10s of microns lateral	Mass-inversion (topological), waveguide confinement 100 nm vertical, few 100 nm lateral
**Q-factor**	High, 10^3 - 10^6, strongly dependent on the material system	Moderate, 10^2 to few 10^3 in experiment	Moderate, 10^2, 150-200 experimentally realised in this work, up to few 10^3 possible theoretically
**FSR Scaling**	$\propto 1/L$, size-dependent	$\propto 1/L$, size-dependent	size-independent, gap-limited
**Robustness**	Low, sensitive to disorder	Moderate to high, sensitive to absorption in the mirrors and layer thickness/composition imperfections	Very high, topologically protected
**Integration of 2D materials and coupling type**	Difficult, surface integration as PhCs are made in suspended membranes or in Si-on-insulator limiting applicability in VIS/NIR; evanescent coupling to deposited 2D material	Vertical stacks, multilayer structures, best integration results in bulky devices with a suspended top mirror	Monolithic van der Waals integration ensuring maximum overlap with the photonic mode

VIII. SUPPLEMENTARY NOTE 8

We note that gold substrate has a weak effect on the PL quenching of the WSe_2 monolayer presented in Fig.6 of the main manuscript, as the monolayer is separated from the gold by a thick WS_2 (69 nm) and is further clad by two hBN layers. This prevents not only the PL quenching due to gold, but also the charge transfer to the surrounding WS_2, the latter being the strongest cause of the PL quenching, see a pioneering paper on this topic for MoS_2/WS_2 heterostructure made from monolayer TMDs in Ref.[8].

The importance of charge transfer between WSe_2 and WS_2 was confirmed in our experiments by fabricating an initial structure in which the WSe_2 monolayer was not encapsulated by hBN layers. In that structure, much lower PL brightness was observed compared with the results reported in Fig.6 in the main text. The results for the WS_2 structure with unencapsulated WSe_2 monolayer is shown in Fig.S6.

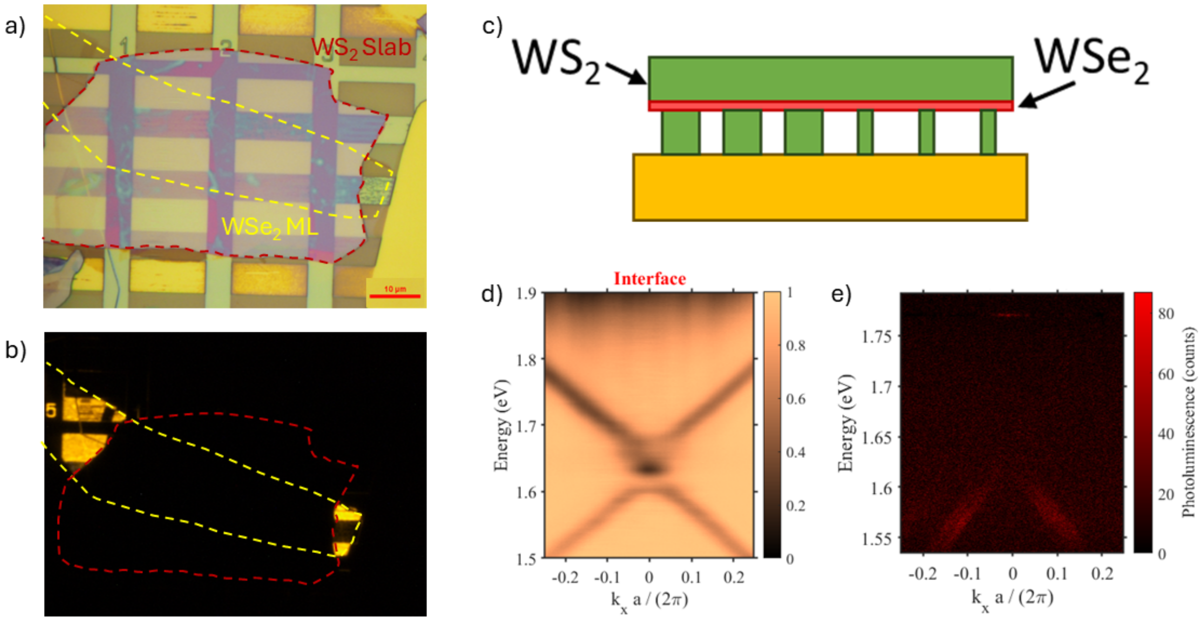

FIG. S6. **WS_2 photonic heterostructure with embedded unencapsulated WSe_2 monolayer.** **(a)** Optical microscope image of the grating heterostructure. Gratings are visible through the thin top WS_2 slab as brighter areas. **(b)** PL image of the grating heterostructure. PL from the monolayer (orange) is strong only outside the top slab and only in the areas where the monolayer is in contact with gratings. PL is strongly supressed where WSe_2 is in contact with planar (unetched) WS_2. **(c)** Schematic of the grating heterostructure with patterned bottom WS_2 creating a double grating interface and a WSe_2 monolayer inserted between the top WS_2 slab and the grating. **(d)** Angle-resolved reflectance contrast measured at the interface between the two gratings with a clearly visible Jackiw-Rebbi around 1.63 eV. **(e)** Angle-resolved PL measured for the same grating as in **(d)**. Strong PL quenching is evident.

IX. SUPPLEMENTARY NOTE 9

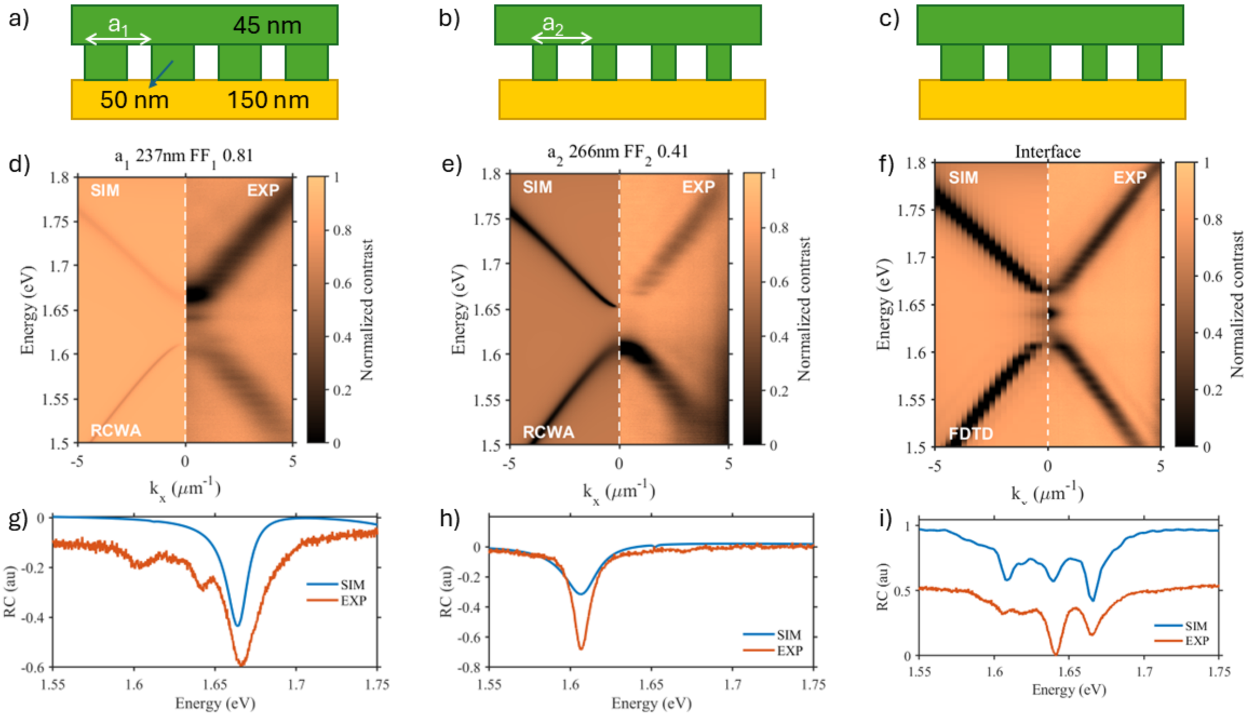

FIG. S7. **Comparison of calculated and experimental data.** The parameters measured using AFM are: grating thickness 47 nm, slab thickness 41 nm, a_1 = 279 nm, FF_1 =0.81, a_2 = 319 nm, FF_2 =0.42. The simulations are performed using RCWA with the following parameters to match the experimental observations: grating thickness 50 nm, slab thickness 45 nm, a_1 = 237 nm, FF_1 =0.81, a_2 = 266 nm, FF_2 =0.41. Overall, the shift of the modes that we must achieve by adjusting these parameters is of the order of 30-50 meV, which may be accounted for by the change of the refractive index around 3%. We note this parameter adjustment procedure most strongly affects the absolute energies of the band edges and the curvature of the dispersion, but not the magnitude of the bandgap itself. (a)-(c) Schematics of the structures simulated and measured in (d)-(i) including two gratings with a high (a) and low (b) filling factors (FFs), and the structure containing an interface between the same gratings. The parameters of the gratings are chosen such that their band structures are inverted and therefore they have different topology. (d)-(f) Calculated (left) and measured reflectance contrast spectra for the structures in (a)-(c), respectively. Note that the widths of the photonic modes depend on the size of the grating measured in the experiment: the smaller is the total size of the grating probed, the wider are the mode peaks due to the increased uncertainty of the wavevector. For the results shown in (d), where mode peaks are wide, the size of that structure was limited by the relatively small overlap between the top WS_2 slab and the etched grating, whereas for the structure in (f) the overlap extended over at least 10 micron x 10 micron. In the calculations, for each grating we use infinite number of grooves, while 20 periods on each side of the boundary is used to calculate the dispersion in (f) where the edge state is clearly visible. (g)-(i) Spectra for $k = 0$ extracted from (d)-(f), respectively. The simulated curves are shown with blue, while the experimentally measured spectra are shown with red.

[1] H. Sigurðsson, H. C. Nguyen, and H. S. Nguyen, Dirac exciton–polariton condensates in photonic crystal gratings, Nanophotonics **13**, 3503 (2024).

[2] L. Lu, Q. Le-Van, L. Ferrier, E. Drouard, C. Seassal, and H. S. Nguyen, Engineering a light–matter strong coupling regime in perovskite-based plasmonic metasurface: quasi-bound state in the continuum and exceptional points, Photon. Res. **8**, A91 (2020).

[3] K. Y. Lee, K. W. Yoo, Y. Choi, G. Kim, S. Cheon, J. W. Yoon, and S. H. Song, Topological guided-mode resonances at non-Hermitian nanophotonic interfaces, Nanophotonics **10**, 1853 (2021).

[4] S. I. Azzam and A. V. Kildishev, Photonic bound states in the continuum: From basics to applications, Advanced Optical Materials **9**, 2001469 (2021).

[5] H. M. Doeleman, F. Monticone, W. den Hollander, A. Alù, and A. F. Koenderink, Experimental observation of a polarization vortex at an optical bound state in the continuum, Nature Photonics **12**, 397–401 (2018).

[6] B. Munkhbat, P. Wróbel, T. J. Antosiewicz, and T. O. Shegai, Optical constants of several multilayer transition metal dichalcogenides measured by spectroscopic ellipsometry in the 300–1700 nm range: high index, anisotropy, and hyperbolicity, ACS Photonics **9**, 2398 (2022).

[7] F. Keilmann and R. Hillenbrand, Near-field microscopy by elastic light scattering from a tip, Philosophical Transactions of the Royal Society of London. Series A: Mathematical, Physical and Engineering Sciences **362**, 787 (2004).

[8] X. Hong, J. Kim, S.-F. Shi, Y. Zhang, C. Jin, Y. Sun, S. Tongay, J. Wu, Y. Zhang, and F. Wang, Ultrafast charge transfer in atomically thin mos_2/ws_2 heterostructures, Nature Nanotechnology **9**, 682 (2014).
